# Case Report: Metachronous colon metastasis of clear-cell renal cell carcinoma seven years after radical nephrectomy

**DOI:** 10.3389/fonc.2026.1948977

**Published:** 2026-10-28

**Authors:** Yuhang Tian, Xinchun Wu, Yongbai Li, Zhidong Gao, Kewei Jiang, Yingjiang Ye, Yichao Yan

**Affiliations:** 1 Department of Gastrointestinal Surgery, Peking University People’s Hospital, Beijing, China; 2 Department of General Surgery, Guizhou Provincial People’s Hospital, Guiyang, China; 3 Department of Gastrointestinal Surgery, Peking University International Hospital, Beijing, China

**Keywords:** case report, clear-cell carcinoma, colon metastasis, literature review, renal cell carcinoma

## Abstract

Colon metastasis from renal cell carcinoma (RCC) is rare and can remain diagnostically elusive when the lesion is predominantly intramural. In this case, we present a 66-year-old man with melena and anorexia. He had undergone left radical nephrectomy for stage I clear-cell renal cell carcinoma (ccRCC) seven years earlier. Computed tomography showed a 5.2 × 4.0 cm mass in ileocecal colon, causing ileal obstruction, with multiple pulmonary nodules. Serum CEA, CA 19-9, and AFP levels were within normal ranges. Colonoscopy described a friable hemorrhagic lesion near the ileocecal valve, but six biopsy specimens from two examinations showed only necrotic tissue. PET-CT findings supported malignancy, and right hemicolectomy was performed. Pathology identified a 5.5 × 5.0 cm clear-cell malignant tumor with full-thickness intestinal wall infiltration. Immunohistochemistry supported metastatic clear-cell RCC. No abdominal recurrence was detected one year later, but pulmonary nodules progressed and the patient died of pulmonary embolism two years after colectomy. This case underscores the need to consider metachronous RCC metastasis in bleeding or obstructing colonic masses after nephrectomy, even when endoscopic biopsy and serum tumor markers are non-diagnostic.

## Introduction

Renal cell carcinoma (RCC) accounts for approximately 3% of adult malignancies and occurs more often in men, with peak incidence in the sixth to seventh decades of life (1, 2). Clear-cell RCC is the dominant histologic subtype, representing about 60-80% of RCC cases (3). At initial diagnosis, approximately 20-30% of patients already have metastatic disease (4). Nephrectomy can be curative for localized RCC, but recurrence after complete resection is not uncommon: 20-40% of patients may develop relapse after R0 surgery, and about 10% of recurrences occur more than five years after nephrectomy (5, 6).

The usual metastatic sites include the lung, liver, bone, lymph nodes and brain (7). Gastrointestinal involvement is much less common, and clinically recognized colonic metastasis is particularly rare. In Saitoh’s autopsy series of 1,451 patients with renal adenocarcinoma, intestinal metastases occurred as part of disseminated disease, but isolated intestinal metastasis was not identified (7). This rarity creates a practical diagnostic problem: a colonic mass in a patient with previous RCC may be interpreted first as primary colorectal cancer, inflammatory disease, or another metastatic tumor, especially when endoscopic biopsy is negative.

In this case, we present a metachronous ileocecal colon metastasis of clear-cell RCC seven years after radical nephrectomy. The novelty of this report lies in its diagnostic and therapeutic relevance: normal serum tumor markers and repeated non-diagnostic colonoscopic biopsies did not exclude malignancy, and preoperative uncertainty regarding primary colorectal cancer justified oncologic colectomy. To further characterize the clinical features and outcomes of this uncommon metastatic pattern, we also review 28 previously reported patients with RCC metastasis to the colon.

## Case presentation

A 66-year-old man presented with tarry stools, anorexia and unintentional weight loss of 4 kg over four weeks. Seven years earlier, he had undergone left radical nephrectomy for stage I (T1N0M0) clear-cell RCC, followed by one month of interferon therapy. Immunohistochemistry of the nephrectomy specimen showed vimentin(+), CD10(+), CK7(-), p53(+), Ki-67 (<20%). No relevant family history was reported.

Physical examination revealed a mobile, irregular 4.0 × 5.0 cm mass in the right lower abdomen on deep palpation. Fecal occult blood testing was positive. The main serum tumor markers, including carcinoembryonic antigen (CEA), carbohydrate antigen 19-9 (CA 19-9), and alpha-fetoprotein (AFP), were within the normal ranges. Computed tomography demonstrated a 5.2 × 4.0 cm mass in the right colon, causing obstruction of the terminal ileum, together with multiple pulmonary nodules (**Figure 1A**). Colonoscopy described a friable, lobulated and easily bleeding lesion near the ileocecal valve (**Figure 1B**). Six biopsy specimens were obtained across two colonoscopic examinations, but histology showed necrotic tissue without diagnostic malignancy. PET-CT showed findings consistent with malignant disease in the intestinal mass (**Figure 1C**).

**Figure 1:**
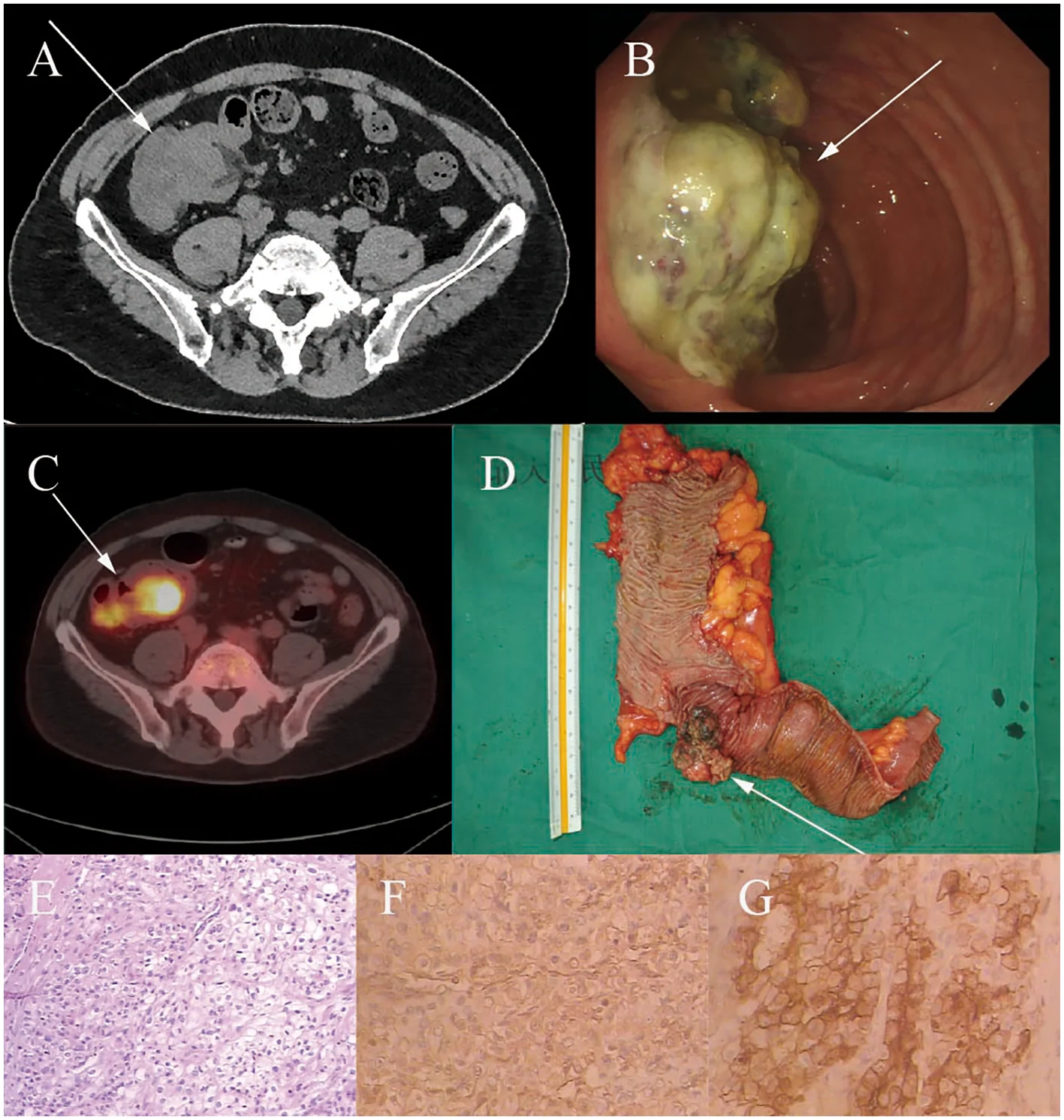
Clinical imaging, endoscopic appearance, PET-CT findings, and gross specimen of ileocecal colon metastasis from clear-cell renal cell carcinoma. **(A)** Abdominal computed tomography shows a 5.2 × 4.0 cm mass in the right colon (white arrow). **(B)** Colonoscopy reveals a friable, lobulated, and hemorrhagic lesion near the ileocecal valve (white arrow). **(C)** PET-CT demonstrates increased fluorodeoxyglucose uptake in the intestinal lesion, supporting malignancy (white arrow). **(D)** Gross specimen after right hemicolectomy shows a variegated gray-yellow mass protruding into the intestinal lumen, located 15 cm from the proximal colonic margin (white arrow). **(E)** Hematoxylin and eosin staining shows tumor cells with clear cytoplasm, consistent with clear-cell morphology (×200). **(F, G)** Immunohistochemical staining shows positivity for vimentin (F, ×400) and CD10 (G, ×400).

Given the patient’s incomplete intestinal obstruction and recurrent hematochezia, laparotomy was performed. A firm, rubbery mass was identified in the right colon. No intra-abdominal metastatic deposits or enlarged lymph nodes were found. Because primary colorectal carcinoma could not be excluded and the lesion involved the ileocecal region, right hemicolectomy was performed rather than limited resection. The pathology report described a specimen containing terminal ileum and ascending colon. Upon opening the specimen, a 5.5 × 5.0 cm variegated golden-yellow, red, and tan mass was identified 15 cm from the proximal colonic margin, protruding into the intestinal lumen (**Figure 1D**). Microscopy showed a clear-cell malignant tumor compatible with metastatic clear-cell RCC (**Figure 1E**). The tumor infiltrated the full thickness of the intestinal wall. Proximal and distal margins were negative. Lymph nodes from multiple pericolic and vascular stations were negative, and no tumor was identified in the omentum. Immunohistochemistry showed CK(+), vimentin(+), CD10(+), PAX8(+), GATA-3(+/-), S-100(-), HMB45(-), MelanA(-), CD117(-), DOG1(-), Ki-67 (30%) (**Figures 1F, G**). Surgical intervention for the pulmonary nodules and postoperative systemic therapy were not pursued because of the patient’s age and his family’s preference.

PET-CT one year after surgery showed no abdominal recurrence, but pulmonary nodules had increased and were considered suspicious for metastases. He remained free of gastrointestinal symptoms and died of pulmonary embolism two years after colectomy. The detailed and chronological clinical course is summarized in **Table 1**.

**Table 1:** Timeline of the clinical episode.

Time	Clinical episode
7 years before presentation	Left radical nephrectomy for stage I clear-cell renal cell carcinoma; interferon therapy for 1 month.
4 weeks before presentation	Tarry stools, anorexia, and 4 kg weight loss.
At presentation	Mobile 4 × 5 cm right lower abdominal mass; fecal occult blood positive.
January 28, 2016	Colonoscopy showed a friable hemorrhagic mass near the ileocecal valve; biopsies showed necrotic tissue without malignancy.
February 16, 2016	CT showed a 52 × 40 mm right-colonic/ileocecal mass with terminal ileal obstruction and multiple pulmonary nodules.
Before surgery	PET-CT suggested malignant intestinal lesions.
February 23, 2016	Right hemicolectomy for recurrent hematochezia and incomplete intestinal obstruction.
After surgery	Pathology and immunohistochemistry confirmed metastatic clear-cell renal cell carcinoma. No surgery for the pulmonary nodules or systemic targeted therapy was performed.
1 year after colectomy	PET-CT showed progression of pulmonary nodules.
2 years after colectomy	Death from pulmonary embolism.

CT, computed tomography; PET-CT, positron emission tomography-computed tomography.

## Discussion

A literature search of PubMed and China National Knowledge Infrastructure was performed to identify previously reported cases of renal cell carcinoma (RCC) metastasis to the colon. Search terms included combinations of “renal cell carcinoma,” “clear-cell renal cell carcinoma,” “colon,” “colonic,” “colorectal,” “metastasis” and “case report,” with equivalent Chinese terms used for the Chinese database. No language restriction was applied. Case reports or case series were included if they described pathologically confirmed cases of RCC metastasis to the colon. This search identified 28 cases published between 1991 and 2025; together with the present case, 29 patients were included for analysis (**Table 2**) (8–33). A major strength of this report is that this structured review allows the present case to be interpreted in a broader clinical context, particularly regarding diagnostic pitfalls, dissemination pathways, and surgical decision-making.

**Table 2:** Clinicopathological characteristics and prognosis of patients with colon metastasis from renal cell carcinoma.

No.	Year	Age/Sex	Primary RCC side	RCC type	Symptoms	Tumor markers	Colonic site	Interval	Treatment	Survival	Source
1	1991	71/M	Left	Clear-cell adenocarcinoma with papillary features	Altered bowel habits, thin stools, abdominal cramps	NA	Splenic flexure	17 years	Left colectomy	NA	Thomason et al. (8)
2	1991	73/M	Left	Clear-cell RCC	Intestinal obstruction	NA	Transverse colon	11 years	Colostomy; left hemicolectomy; later right hemicolectomy	8 years	Ruiz et al. (9)
3	1996	83/M	Right	Clear-cell RCC, alveolar type	Melena, abdominal mass	CEA, CA19-9, AFP normal	Transverse colon	7 years	Partial transverse colectomy	NA	Tokonabe et al. (10)
4	1998	72/F	Right	RCC, clear-cell morphology	Abdominal pain, right-colon mass	CEA normal	Hepatic flexure	5 years	Right hemicolectomy	NA	Avital et al. (11)
5	2000	73/M	Left	Clear-cell RCC	Hematochezia, melena	NA	Sigmoid colon	8 years	Left colectomy	NA	Diaz-Candamio et al. (12)
6	2001	47/M	Left	RCC with clear cytoplasm and alveolar pattern	Hematochezia, left abdominal pain, fullness, watery diarrhea	CEA, CA19-9, AFP normal	Transverse colon	9 years	Partial transverse colectomy	NA	Utsunomiya et al. (13)
7	2001	68/M	Right	Clear-cell renal adenocarcinoma	Intermittent rectal bleeding	NA	Sigmoid colon	1 year	Left hemicolectomy; IFN-alpha + vinblastine planned	NA	Regenet et al. (14)
8	2002	76/F	Left	Mixed clear and granular cell RCC	Dyspepsia, lethargy, right upper abdominal pain	CEA, CA19-9, AFP normal	Ascending colon	4 years	Interferon	1 year	Lee et al. (15)
9	2006	65/F	Left; contralateral right renal mass	Sarcomatoid RCC; contralateral clear-cell carcinoma also reported	Anemia	NA	Sigmoid colon	1 year	IL-2 + IFN-alpha	3 weeks	Invernizzi et al. (16)
10	2006	78/M	Left	Clear-cell RCC	Loose stool, recurrent hematochezia	NA	Descending colon	30 months	Left hemicolectomy	NA	Li et al. (17)
11	2008	60/M	Right	Clear-cell RCC	Fatigue, dyspepsia, right upper abdominal pain	NA	Hepatic flexure	5 years	Right hemicolectomy	NA	Yetkin et al. (18)
12	2009	50/M	Left	Clear-cell RCC	Intermittent hematochezia, increased stool frequency, incomplete obstruction	NA	Descending colon	18 months	Left hemicolectomy	NA	Wang et al. (19)
13	2010	65/F	Left	Clear-cell RCC	Acute abdominal pain, collapse, intra-abdominal bleeding	NA	Transverse colon	9 years	Emergency local excision	6 years	Jadav et al. (20)
14	2013	63/M	Left	RCC	Irregular stools, bloating	NA	Rectosigmoid colon	2 years 10 months	Left hemicolectomy	NA	Milovic et al. (21)
15	2013	35/M	Left; later right renal lesion	Left RCC with light granular cell; later right clear-cell RCC also reported	Nausea, vomiting, abdominal distension, abdominal tenderness, right testis pain	AFP normal	Ileocecal colon	Within 2 years	Cecal resection; temsirolimus	2.5 years	Milovic et al. (21)
16	2013	39/M	Right	RCC	Right thigh pain, liquid stools, constipation	NA	Ileocecal colon	4 years	Right hemicolectomy; IFN-alpha + 5-FU	9 months	Milovic et al. (21)
17	2016	67/M	Left	Clear-cell RCC	Nausea, vomiting, abdominal distension, hematochezia, decreased stool caliber, obstruction	NA	Rectosigmoid colon	9 years	Sigmoidectomy with anterior resection	6 months	Vo et al. (22)
18	2017	48/F	Right	Clear-cell RCC	Upper abdominal pain	NA	Hepatic flexure	3 years	Palliative ileostomy; sunitinib; later right hemicolectomy	NA	Peng et al. (23)
19	2019	63/M	Left	Clear-cell RCC	Abdominal pain, nausea, hematochezia	NA	Splenic flexure	5 years	Left hemicolectomy with Hartmann’s procedure	6 months	Subasi et al. (24)
20	2020	65/M	Right	Clear-cell RCC	Asymptomatic, screening colonoscopy finding	NA	Ascending colon	6 months	Laparoscopic right hemicolectomy	NA	Kataoka et al. (25)
21	2020	75/M	Left	Clear-cell RCC	Diarrhea, watery hematochezia, left abdominal tenderness	NA	Descending colon	More than 10 years	Left hemicolectomy	NA	Ge et al. (26)
22	2023	40/M	Right	Clear-cell RCC with sarcomatoid features in metastasis	Worsening abdominal pain, low-grade fever	NA	Splenic flexure	Synchronous with renal tumor diagnosis	Segmental colectomy with mesocolic mass resection; pazopanib	22 months	Patidar et al. (27)
23	2023	65/F	Right	Clear-cell RCC	Blood in stool, rectal bleeding	NA	Sigmoid colon	8 years	Sigmoidectomy	32 months	Li et al. (28)
24	2024	58/NA	NA	Clear-cell RCC	Discomfort, fatigue	NA	Unspecified colon	13 years	Laparoscopic-assisted colon tumor resection	NA	Yu et al. (29)
25	2024	40s/M	Right	Clear-cell RCC	Abdominal pain, weakness, melena	NA	Ascending colon	7 years	Hospice care	NA	Lin et al. (30)
26	2024	87/M	Left	RCC, likely clear-cell morphology	Melena, abdominal pain	CEA and CA19–9 normal	Splenic flexure	3 years	NA	NA	Alvi et al. (31)
27	2025	80/M	Left	Clear-cell RCC	Rectal bleeding, weight loss, constipation, hematochezia	NA	Splenic flexure	NA	Endoscopic polypectomy	NA	Canter et al. (32)
28	2025	62/M	Left; later right renal lesions	Clear-cell RCC	Hematochezia with clots, syncope, tissue-like mass per rectum	NA	Descending colon	8 years	Palliative radiation; tivozanib planned	NA	Bravo Thompson et al. (33)
29	2026	66/M	Left	Clear-cell RCC	Melena, anorexia, weight loss, hematochezia, incomplete obstruction	CEA, CA19-9, AFP normal	Ileocecal colon	7 years	Right hemicolectomy	2 years	Present case

RCC, renal cell carcinoma; M, male; F, female; NA, not available or not reported in the source article; CEA, carcinoembryonic antigen; CA19-9, carbohydrate antigen 19-9; AFP, alpha-fetoprotein; IL-2, interleukin-2; IFN, interferon; 5-FU, 5-fluorouracil.

These compiled cases reveal several distinct clinical patterns of this rare metastatic presentation. Sex was reported in 28 of the 29 patients, and 22 of these 28 patients (78.6%) were male. Most colonic metastases showed clear-cell morphology (27/29, 93.1%), whereas sarcomatoid, granular-cell, mixed, or incompletely specified RCC subtypes occurred in only a few cases. Colonic involvement was predominantly metachronous, with a median interval of 5 years and a range from synchronous detection to 17 years after nephrectomy. More than half of the evaluable cases occurred at least five years after primary renal surgery. Primary renal laterality was reported in 28 cases. Among 24 anatomically classifiable cases, 18 (75.0%) showed ipsilateral and 6 (25.0%) contralateral colonic involvement; transverse and unspecified lesions were excluded from this comparison. Metastatic lesions were distributed throughout the colon, including the ileocecal colon, ascending colon, hepatic flexure, transverse colon, splenic flexure, descending colon, sigmoid colon, and rectosigmoid colon. Clinical presentations were nonspecific and most commonly included hematochezia, melena, abdominal pain, or bowel obstruction. Serum tumor marker data were reported in only 7 cases (24.1%), with all available values being within normal ranges. Treatment was predominantly surgical: 23 patients (79.3%) underwent colonic resection, ranging from local or segmental resection to formal colectomy, and one additional patient underwent endoscopic polypectomy (8–33).

A major clinical pitfall highlighted by our case and several cases in the literature is the potential for false-negative endoscopic mucosal biopsies. In our patient, two consecutive colonoscopies with six biopsies yielded only necrotic tissue and acute inflammatory cells, masking the underlying malignancy. This diagnostic failure occurred in multiple reported cases, where repeated colonoscopic biopsies were non-diagnostic, delayed definitive diagnosis, or initially suggested benign ulceration (10, 12, 13, 19, 22, 32). The pathological explanation for this diagnostic challenge lies in the distinct metastatic growth pattern of RCC. Unlike primary colorectal adenocarcinomas that originate from the mucosal epithelium, metastatic RCC cells typically seed hematogenously into the submucosal and muscularis propria layers of the colonic wall (34). As the intramural mass expands, it compromises the blood supply to the overlying mucosa, leading to secondary mucosal necrosis, ulceration, and hemorrhage. Consequently, standard superficial endoscopic biopsies may sample only superficial necrotic debris and inflammatory tissue, rather than tumor cells located deeper within the bowel wall. Clinicians should therefore interpret a negative colonoscopic biopsy cautiously in patients with a history of RCC, and consider repeat sampling, deeper biopsy, or surgical resection when clinical and imaging findings remain suspicious.

Serum tumor markers represent another diagnostic limitation in this setting. In the present patient, CEA, CA 19-9, and AFP were within the normal ranges. Similarly, among the published cases in which both CEA and CA 19–9 were reported, both markers were within the normal ranges despite histologically confirmed colonic RCC metastasis. Although normal CEA and CA 19–9 levels may reduce support for a primary colorectal neoplasm, they do not exclude a malignant colonic lesion, particularly metastatic RCC. Moreover, no established serum tumor marker is available for detecting RCC recurrence. Therefore, in patients with a previous history of RCC and a suspicious colonic lesion, diagnostic decision-making should not rely on serum tumor markers alone, but should integrate the clinical history, imaging findings, endoscopic appearance, and definitive histopathological assessment.

The dissemination pathways and therapeutic management of colonic RCC metastasis require a highly individualized, multidisciplinary approach. To further explore the anatomical relationship between the primary renal tumor and colonic involvement, we extracted the laterality of the primary RCC from the published cases summarized in **Table 2**. Among cases in which both renal laterality and colonic segment could be assessed, ipsilateral involvement appeared to be more frequent; however, contralateral metastases were also observed. Transverse colonic lesions were not classified as either ipsilateral or contralateral unless a flexural location was specified. In our patient, the primary ccRCC was left-sided, whereas the metastasis arose in the right colon, with all regional lymph nodes and omentum free of malignancy. This anatomical distribution argues against direct invasion, nephrectomy-bed recurrence with contiguous extension, or adjacent peritoneal seeding, and instead supports hematogenous dissemination as the most plausible pathway. Hematogenous spread in RCC may occur through retrograde venous flow via the vertebral venous plexus (Batson’s plexus) or through the systemic arterial circulation (7, 12, 33). The concurrent pulmonary nodules suspicious for metastases in our case further support a systemic hematogenous route, in which tumor cells seeded both the lungs and the colonic submucosa (33).

For treatment, surgical resection remains the cornerstone of management. For solitary colonic metastasis from RCC, complete surgical resection may offer durable disease control in selected patients, consistent with evidence that complete metastasectomy is associated with improved survival outcomes (35, 36). In the presence of disseminated systemic disease, such as the progressive pulmonary nodules in our patient, surgical intervention is indicated as a palliative measure to manage acute complications such as intestinal obstruction and recurrent bleeding. While systemic treatments combining immune checkpoint inhibitors and tyrosine kinase inhibitors have significantly improved survival in metastatic clear-cell RCC (37–40), treatment decisions should be tailored to the patient’s performance status, age, and personal preferences. Practically, when a patient with a history of ccRCC presents with a suspicious colonic mass and repeatedly non-diagnostic biopsies, the surgeon should consider resection rather than pursuing further biopsy confirmation; if primary colorectal cancer cannot be excluded, the procedure should follow oncologic colectomy principles.

This report has several limitations. First, the patient did not receive standard postoperative systemic therapy after colectomy, which may have contributed to the subsequent progression of pulmonary nodules and survival outcome. In addition, the literature review was based on published case reports and case series, which are subject to publication bias and incomplete reporting. Therefore, the observed clinical patterns should be interpreted as descriptive rather than definitive.

## Conclusion

Colon metastasis of clear-cell RCC is rare but clinically important. In patients with a history of nephrectomy, a bleeding or obstructing colonic mass should not be dismissed as primary colorectal disease or inflammation solely because endoscopic biopsy is non-diagnostic or serum tumor markers are within normal ranges. Cross-sectional imaging, PET-CT when appropriate, and definitive pathology with immunohistochemistry remain essential for diagnosis. Surgical resection may provide symptom relief and local control, while systemic disease burden should guide broader oncologic management.

## Data Availability

The original contributions presented in the study are included in the article/**Supplementary Material**. Further inquiries can be directed to the corresponding author.
