# Supplementary material for: Case Report: Metachronous colon metastasis of clear-cell renal cell carcinoma seven years after radical nephrectomy
Source: Front Oncol. 2026 Oct 28;16:1948977. doi: 10.3389/fonc.2026.1948977 (PMC13751372; doi:10.3389/fonc.2026.1948977)
Supplement: Supplementary file 1 [file Table1.docx]

**CARE checklist**

| Item | Checklist item | Reported (Yes/No) |
| --- | --- | --- |
| 1 | Title | Yes |
| 2 | Key words | Yes |
| 3a | Introduction: unique case and contribution | Yes |
| 3b | Main symptoms and clinical findings | Yes |
| 3c | Diagnosis, intervention and outcomes | Yes |
| 3d | Conclusion/take-away lesson | Yes |
| 4 | Introduction: why this case is unique | Yes |
| 5a | De-identified patient information | Yes |
| 5b | Primary concerns and symptoms | Yes |
| 5c | Medical/family history | Yes |
| 5d | Previous interventions and outcomes | Yes |
| 6 | Physical examination and clinical findings | Yes |
| 7 | Timeline | Yes |
| 8a | Diagnostic testing | Yes |
| 8b | Diagnostic challenges | Yes |
| 8c | Diagnosis including differential diagnosis | Yes |
| 8d | Prognosis/staging | Yes |
| 9a | Type of therapeutic intervention | Yes |
| 9b | Administration of intervention | Yes |
| 9c | Changes in intervention and rationale | Yes |
| 10a | Clinical outcomes | Yes |
| 10b | Follow-up diagnostic results | Yes |
| 10c | Intervention adherence/tolerability | No |
| 10d | Adverse/unanticipated events | Yes |
| 11a | Strengths and limitations | Yes |
| 11b | Relevant medical literature | Yes |
| 11c | Scientific rationale/conclusions | Yes |
| 11d | Take-away lessons | Yes |
| 12 | Patient perspective | No |
| 13 | Informed consent | Yes |
